# Usability of the Coach-Supported Dementia Prevention App ENHANCE (Tailored Intervention for Brain Health and Cognitive Enrichment) in Older Adults: 1-Week Mixed Methods Study

**DOI:** 10.2196/92800

**Published:** 2026-07-23

**Authors:** Tsz Kiu Clare Yu, Gill Livingston, Richard Boczko, Kealan Forristal, James Jamison, Carl Leckstein, Vrushti Mehta, Hee Kyung Park, Aneesha Singh, Andrew Sommerlad, Sergi G Costafreda

**Affiliations:** 1 Division of Psychiatry, University College London, Maple House, 149 Tottenham Court Road, London, W1T 7NF, United Kingdom, +44 20 3987 2384; 2 North London NHS Foundation Trust, London, United Kingdom; 3 Faculty of Health Sciences, University of Hull, Hull, England, United Kingdom; 4 Social, Genetic & Developmental Psychiatry Centre, King's College London, London, England, United Kingdom; 5 Division of Psychology and Language Sciences, University College London, London, England, United Kingdom; 6 Department of Neurology, Samsung Medical Center, Sungkyunkwan University, Seoul, Republic of Korea; 7 UCL Interaction Centre, University College London, London, England, United Kingdom

**Keywords:** dementia prevention, older adults, mHealth, underserved populations, usability testing, user experience, cognitive training

## Abstract

**Background:**

Multidomain dementia-prevention interventions delivered via apps have the potential to reach large populations. However, existing trials have tended to recruit more socioeconomically advantaged participants, raising concerns that the resulting interventions may be less usable for older adults from minority ethnic, lower educational, or lower socioeconomic backgrounds, who are at higher risk of dementia. The Tailored Intervention for Brain Health and Cognitive Enrichment (ENHANCE) app was designed to address this by prioritizing accessibility and engagement across diverse user groups.

**Objective:**

This study evaluated the usability and user experience of the ENHANCE prototype during a 1-week at-home supported-use test and explored factors influencing use and engagement among older adults.

**Methods:**

We purposively recruited adults aged 60‐80 years without dementia for a 1-week mixed methods usability evaluation through community settings, including groups underrepresented in dementia-prevention trials. Participants had at least 1 of 10 prespecified dementia risk factors, attended a face-to-face onboarding session with a coach, used the app at home for 7 days with ongoing coach support, and completed a posttest interview and satisfaction survey. We analyzed quantitative data, including app usage metrics and survey responses descriptively, and used reflexive thematic analysis of qualitative data from onboarding sessions, posttest interviews, coaching calls, and in-app messages.

**Results:**

Ten participants participated in the study. The mean age was 68 (SD 6) years, and 7 were females. Participants represented a wide range of deprivation (Index of Multiple Deprivation deciles: mean 4, SD 2, range 1‐8), with 6 from ethnic minority backgrounds. All met prespecified minimum-use targets (watching a module video, completing a check-in, and playing assigned games at least once). Many demonstrated additional voluntary engagement: 5 rewatched the risk factor video, 7 used the in-app messaging feature, and among the 5 participants with the hypertension module, blood pressure was logged on an average of 5 out of 7 days. Survey responses indicated high satisfaction, perceived usefulness, and ease of use; 9 participants intended to continue using the app and would recommend it to peers. Qualitative analysis identified engagement facilitators, including rewarding game design, familiar interfaces, appropriately challenging gameplay, consistent virtual rewards, trusted expert information combined with peer stories, and coach support. Barriers included unclear visual cues, insufficient accommodation of motor or sensory impairments, and visual discomfort in some games.

**Conclusions:**

Older adults found the ENHANCE prototype usable, acceptable, and engaging over 1 week. Human coaching, inclusive design, and integration of expert and peer narratives were highlighted as key engagement drivers. These findings support further feasibility testing to examine longer-term engagement and provide design insights for more inclusive digital health interventions.

## Introduction

The number of people with dementia globally is expected to reach 152 million by 2050, posing a major challenge for individuals, families, and health and social care systems [1]. As a result, prevention is a public health priority. The Lancet Commission on dementia prevention, intervention, and care estimates that 45% of cases could be prevented by eliminating 14 potentially modifiable risk factors, including less education, hypertension, diabetes, physical inactivity, smoking, obesity, depression, hearing loss, excessive alcohol, and social isolation [2]. Multidomain interventions addressing several dementia risk factors simultaneously can provide cognitive benefits for older adults at risk, though effects are typically modest [3,4]. For example, a network meta-analysis of over 100 RCTs found small improvements in cognition [3], while the large “Maintain Your Brain” randomized controlled trials (n>6000) reported modest gains over 3 years in the online multidomain lifestyle intervention [4,5].

People with lower socioeconomic status and those from ethnic minority groups are at greater risk of developing dementia likely due to higher risk factor prevalence [6-10]. Despite being at higher risk, these groups are often inadequately represented or reported in prevention trials [11-13]. In the Advanced Cognitive Training for Independent and Vital Elderly (ACTIVE) cognitive training trial (n=2832), participants were disproportionately highly educated (88.6% high school diploma+ vs 67% in the US population aged 65 y and older), healthier (higher health scores in health survey), and less hospitalized than national norms [14]. Similarly, in the Finnish Geriatric Intervention Study to Prevent Cognitive Impairment and Disability (FINGER) trial (a 2-year multidomain intervention; n=1260), participants had higher education levels and were younger or healthier than nonscreened eligible individuals from the same population [15]. These patterns underscore the need for more inclusive dementia prevention strategies that target underserved communities. Given the elevated baseline risk in these populations, even modest improvements in risk factor profiles could yield substantial reductions in dementia incidence at the population level.

In this paper, we use the term “underserved” to refer to groups who may experience higher dementia risk but face barriers to accessing and engaging with prevention interventions and who are often underrepresented in dementia prevention research. This includes (but is not limited to) older adults living in more socioeconomically deprived areas, with lower levels of education, and those from ethnic minority backgrounds.

Digital health interventions, particularly app-based approaches, offer a promising approach to more inclusive dementia prevention as they provide remote access to content, potentially benefiting those with less mobility, in rural areas, or facing financial barriers to in-person programs [16]. Apps can also support multiple languages, improving accessibility for ethnic minority older adults [17].

App use is increasingly common among older adults, including those from low-income groups in high-income countries. In the United Kingdom (2021‐2022), 81% of adults aged older than 65 years used the internet, with 49% of these older adults accessing it via tablet [18]. Moreover, 67% of individuals from lower socioeconomic backgrounds across all age groups owned a smartphone—a figure likely to have risen due to increased digital adoption during the COVID-19 pandemic [19]. Importantly, a recent study suggests that device access can translate into engagement; individuals from underserved populations use health apps at rates comparable with more advantaged groups and derive similar benefits [20]. However, usability challenges and lower digital confidence remain significant barriers to long-term engagement, especially among this population [21,22]. Addressing these through inclusive design is essential for long-term engagement and use.

To address these gaps, we developed Tailored Intervention for Brain Health and Cognitive Enrichment (ENHANCE), a theory-informed, coach-supported, app-based intervention targeting modifiable dementia risk factors in older adults. A central aim of ENHANCE was to create an intervention that is accessible and usable across the whole older population, including the underserved people. The development process was centered on co-design with community-recruited participants, with a particular focus on individuals underrepresented in dementia prevention research, including those with lower educational attainment, from ethnic minority backgrounds, and with lower socioeconomic status. We also collaborated closely with patient and public involvement representatives and experts in human-computer interaction. Usability testing and iterative refinement were conducted specifically with older adults with lower education and socioeconomic status (refer to our previous work for detailed description) [23].

This study aims to evaluate user experience with the ENHANCE prototype over a 1-week period, identifying key facilitators and barriers to use.

This study addresses the following research questions:

1. What are the experiences of older adults using ENHANCE over 1 week?
2. What factors facilitate or hinder at-home, 1-week use and engagement with the intervention?

## Methods

### Overview

**Figure 1.**
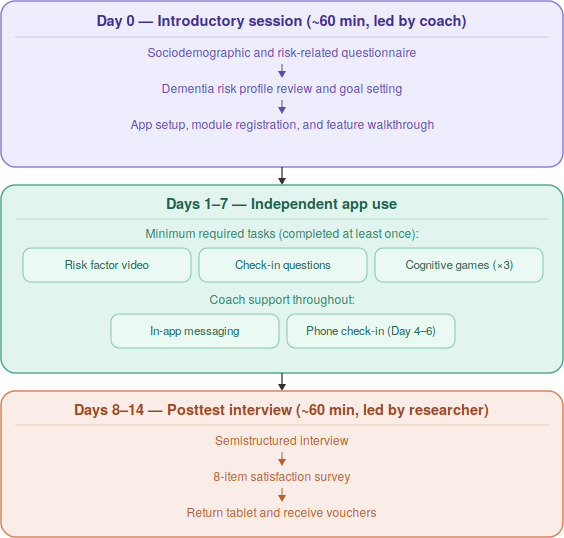
Study’s flowchart.

We conducted a 1-week usability test of the ENHANCE app prototype with older adults, including individuals from underserved backgrounds in the United Kingdom, using a mixed methods approach. Recruitment and data collection took place in London between November 2024 and February 2025. An overview of the study procedure is presented in Figure 1.

This project is part of the ENHANCE study, which will evaluate the feasibility, acceptability, and effectiveness of the ENHANCE intervention in reducing dementia risk among healthy older adults from underserved backgrounds.

### Ethical Considerations

The Institutional Review Board of University College London (ID 24235/001) gave ethical approval on June 19, 2023.

Before enrollment, all participants received a participant information sheet detailing the study purpose, investigator names, study procedures, and the expected time commitment (60-min introductory session, 7 days of independent app use, 60-min posttest interview, and a 5-min satisfaction survey). Participants were informed of their right to withdraw at any time without consequence and gave written informed consent before any data collection. All research participation was voluntary.

We pseudonymized collected data using alphanumeric codes. The code list and consent forms were stored separately from the anonymized data in locked cabinets (paper records) and on a password-protected, encrypted institutional drive (electronic records), accessible only to the core research team. Data are retained for 1 year following completion of the relevant analysis.

### Eligibility Criteria and Recruitment

Participants were older adults residing in the United Kingdom. Eligibility criteria were as follows:

- Participants were included if they (1) were aged between 60 and 80 years; (2) had at least 1 dementia risk factor targeted by the ENHANCE intervention (<8 y of full-time education, diagnosed diabetes, hypertension, BMI>30kg/m^2^, current smoker, >21 units of alcohol/week, <2.5 h of exercise/week, depressive symptoms identified using 2 adapted screening questions as per Patient Health Questionnaire−2 [PHQ-2] [24], social isolation assessed with 1 adapted question based on the Lubben Social Network Scale [25], and self-perceived hearing impairment); (3) were able to read and communicate in basic English; and (4) had the mental capacity to provide informed consent.
- Participants were excluded if they reported a dementia diagnosis or any disability that would significantly impact tablet use, both assessed via self-reported screening questions (Multimedia Appendix 1).

We predetermined a sample size of 10 participants, based on guidance suggesting that this number can identify approximately 95% of major usability issues [26]. We did not set eligibility criteria based on education, ethnicity, or area deprivation; instead, we used a recruitment strategy designed to reach underserved groups. Participants were drawn from a database of over 100 individuals (aged 60 years or older) recruited through community settings serving underserved populations in London, including food banks, neighborhood shopping malls, religious groups, and community organizations supporting low-income and ethnic minority communities. No centers serving people with dementia were included. Participants had previously taken part in ENHANCE coproduction activities [23] and had provided consent to be contacted for future research.

To inform purposive sampling, we extracted participants’ age, ethnicity, and Index of Multiple Deprivation (IMD) scores from self-reported data collected in the previous study [23]. The IMD is a UK-based measure of relative deprivation derived from participants’ postcodes and divided into deciles, with lower scores indicating higher levels of deprivation [27]. This information was used to purposefully recruit individuals from more deprived areas and non-White backgrounds.

TKCY, a PhD student with 6 years of dementia research experience, recruited potential participants by phone, email, or text. She informed them that no previous digital skills or home access to a tablet were required. Interested individuals completed a brief telephone screening for eligibility, covering age, dementia diagnosis status, as well as dementia health and lifestyle risk factors (Multimedia Appendix 1).

### The ENHANCE Intervention

ENHANCE is a coach-supported, app-based intervention designed to help older adults aged from 60‐80 years, including those from the underserved background, reduce dementia risk by addressing 10 modifiable risk factors—hypertension, diabetes, physical inactivity, obesity, depression, smoking, excessive alcohol use, social isolation, hearing loss, and low education—through lifestyle behavior change and cognitive engagement.

The app’s features fall into 3 categories:

- Core content: 7 cognitive training games; modules for 9 other risks which include educational videos and brief weekly check-in questions to support motivation and self-monitoring for lifestyle change tailored to participants’ selected risk factors.
- Engagement tools: A unifying “meadow” home screen and rewards page where participants can use virtual seeds to plant flowers in their meadow for playing games, watching videos, and answering check-ins; and a progress tracker showing weekly stars earned from completed activities.
- Human support: Scheduled coaching sessions (45 min in-person onboarding session and fortnightly remote short follow-ups); a coach-only dashboard to monitor participants’ activities; in-app messaging with the coach; and optional family involvement.

**Figure 2.**
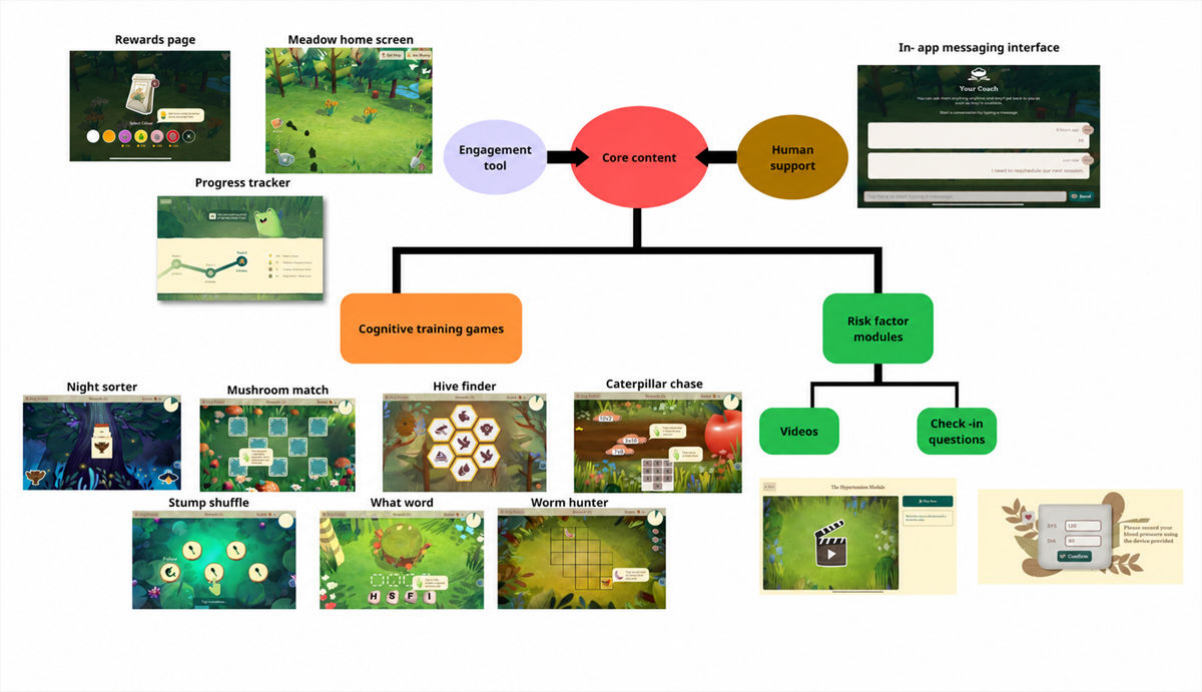
Screenshots of the ENHANCE app.

All participants, regardless of their educational attainment, were given access to the cognitive training games in the ENHANCE intervention as they are rewarding in themselves. For the remaining 9 risk factors, each participant selected 1 risk factor module to focus on during the 1-week testing period. Figure 2 illustrates screenshots from the ENHANCE app. Further details about the app design are reported elsewhere [23].

### Procedures

#### Overview

The 1-week usability testing consisted of three components: (1) an introductory session on day 0, (2) independent app use from days 1 to 7, and (3) a posttest feedback interview conducted between days 8 and 14.

#### Introductory Session (Day 0)

Each participant attended a 60-minute introductory session about the app either at the university or at their home, choosing either. A coach (KF, CL, or VM) with an MSc in Psychology who contributed to the coaching manual development led the session, while the researcher (TKCY), a member of the research team who developed the ENHANCE app, observed participants’ engagement with both the app and the coach.

The session began with participants completing a brief sociodemographic and risk-related questionnaire (Multimedia Appendix 2), by answering about age, post code (to derive the IMD to measure socioeconomic status), educational level, and key lifestyle and health factors relevant to dementia risk. Based on responses, the coach reviewed each participant’s dementia risk profile, discussed the identified risk factors and which one they wished to target during the 1-week testing, and supported the creation of a SMART (specific, measurable, achievable, relevant, time-bound) goal [28] for positive behavior change related to this risk factor. The coach then guided participants through app setup, registration for the selected module, and key features, encouraging questions throughout.

At the end of the session, participants were given a tablet with the app installed and a SIM card and reminded to complete at least 1 video, 1 check-in tool, and each of the 3 cognitive training games at least once during the week; other activities (eg, additional gameplays and video rewatch) were optional.

The session was audio-recorded, and TKCY took notes on participants’ interactions with the app, the coach, and any observed difficulties or misunderstandings.

#### App Testing Week (Day 1-Day 7)

Participants used the app independently for 7 consecutive days. On the app, each participant received:

- 3 randomly assigned cognitive training games from the games on the app. These covered different areas of cognition and could each be played up to 10 times per week after first completion
- 1 video from their selected module (rewatchable without limits)
- Risk factor–specific weekly check-in questions (eg, number of days exercise was completed in the previous wk, with daily logging enabled only for the blood pressure module).

Minimum required use was defined as completion of three core tasks during the testing period: (1) watching the assigned risk factor video at least once, (2) responding to the check-in question at least once, and (3) playing each of the 3 assigned games at least once.

Coach support was available throughout the testing week. Participants could message their coach via the in-app messaging feature. They also received a phone check-in on days 4‐6. The check-in call was audio-recorded for further analysis.

#### Posttest Feedback Interview (Day 8-Day 14)

TKCY conducted and audio-recorded a posttest interview (approximately 60 min) at the same venue as the introductory session, within 1 week after app use, to gather feedback on participants’ experiences with the ENHANCE app and coaching. No previous relationship existed between TKCY and the participants before study commencement. Participants were informed that TKCY was a PhD student involved in the project as part of her doctoral study.

TKCY used a semistructured guide co-developed by the core research team and patient and public involvement representatives (Multimedia Appendix 3). The interview covered participants’ general impressions, a section-by-section app review for detailed feedback, reflections on the coaching experience, and any potential barriers. TKCY reviewed app usage data in advance to tailor questions, particularly around unused features.

Immediately following the posttest interview, participants completed an 8-item, 1-page satisfaction survey taking approximately 5 minutes on a laptop preloaded with a direct Qualtrics link. To minimize social desirability bias, participants completed the survey independently, with TKCY briefly demonstrating the navigation and submission and then withdrew. All 8 items were mandatory, and Qualtrics was configured to prevent submission unless all items were completed. Participants then saw a review screen summarizing their responses, allowing them to amend answers if needed.

TKCY developed the satisfaction survey from the Client Satisfaction Questionnaire (CSQ-8) [29]—a validated measure of programs and services satisfaction. She adapted it for ENHANCE as no existing tool adequately captured the app-based gameplay, dedicated risk factor modules, and one-to-one health coaching. SGC and a member of the Patient and Public Involvement team provided feedback on wording, clarity, and coverage of the key ENHANCE components, and TKCY iteratively refined the survey. The final survey assessed app satisfaction, game enjoyment, perceived behavior-change potential of the risk factor modules, and coaching satisfaction using a 5-point Likert scale (Multimedia Appendix 4). Participants then returned the tablet and received £80 (£1=US $1.34) vouchers for their time (£25 for onboarding, £25 for postsession feedback, and £30 for app use).

### Analyses

All quantitative data were analyzed using SPSS (version 25; IBM), and qualitative data were analyzed using NVivo 14 (Lumivero).

#### Demographic Data

Demographic data collected via a brief questionnaire administered during the introductory session were analyzed descriptively, with means, SDs, and range reported for continuous variables and counts for categorical variables.

#### Quantitative Data

##### App Use (Minimum and Additional App Use)

App use data were extracted from the backend following the app testing week and analyzed to (1) assess whether participants met prespecified minimum-use targets during the testing period and (2) characterize additional patterns of engagement beyond these targets.

Additional app use was assessed using 4 indicators. First, the number of times the risk factor video was rewatched was obtained from backend logs. Second, only participants assigned to the hypertension module had access to respond to the check-in more than once during the study week. Additional responses to the check-in question=(total number of check-in days÷total days of app use)×7. Third, additional gameplay beyond the minimum requirement=(total number of plays÷total days of app use)×7. The values were then averaged across all participants to yield the mean weekly gameplay frequency for each game. Finally, use of the in-app messaging feature at least once via backend data. Participants were coded as having used the feature if they initiated or responded to at least 1 message with the coach. Those with no interaction were coded as nonusers.

##### Posttest App Satisfaction Survey

Responses to 8 posttest survey questions were analyzed descriptively and summarized as counts for each response option.

### Qualitative Data

All audio recordings were transcribed using data regulation-compliant software (Happy Scribe [Happy Scribe Ltd]) and reviewed by TKCY for accuracy and then imported into NVivo 14. TKCY analyzed the data using 6-step reflexive thematic analysis by Braun and Clarke [30,31]. She also maintained a reflexive journal to consider how her background might have influenced the interviews and analysis. She used the COM-B (capability, opportunity, motivation—behavior) model as a guiding framework to identify facilitators and barriers influencing app engagement [32] to provide a comprehensive overview of internal and external factors influencing behavior. It has been widely applied in studies understanding app use and user engagement [32-35] and aligns well with qualitative interview-based data analysis.

- Capability refers to an individual’s ability to carry out the behavior, which includes psychological factors (such as knowledge, memory, and cognitive skills) and physical abilities (such as physical strength or motor skills).
- Opportunity encompasses the external circumstances that facilitate or encourage the behavior. These may be physical (eg, time availability and resources) or social (eg, social support or peer influence).
- Motivation involves the internal processes that initiate or maintain behavior. This includes reflective motivation (like conscious goals, intentions, and evaluations) and automatic motivation (such as emotions, desires, and habits).

She analyzed each qualitative data source separately, generating initial codes and developing them into candidate themes with theme refinement carried out through 5 biweekly meetings between TKCY, A Singh, GL, SGC, and HKP. As consistent patterns emerged, findings from each qualitative data source were integrated into a unified set of themes at the end.

### Reflexivity Statement

TKCY is a female PhD student in her 30s from Hong Kong with a background in psychology and research experience in aging and dementia research. She is a core member of the team that co-designed the ENHANCE app. Her role in the project may have influenced how she conducted interviews and interpreted data. Her familiarity with app usability literature may also have shaped how she identified themes during coding. As a young, educated researcher proficient in technology, she acknowledged a risk that she may underestimate usability challenges and overlook barriers faced by older adults. To mitigate this, she remained open to participants’ perspectives, actively probed for any concerns raised by participants during interviews, and regularly discussed her reflections with supervisors throughout the process.

### Data Integration Between Quantitative and Qualitative Data

TKCY analyzed quantitative and qualitative data separately, then integrated them through collaborative discussions with the research team of A Singh, GL, SGC, and HKP. These discussions involved comparing and contrasting findings across data sources to identify areas of convergence and divergence.

## Results

### Overview

All 10 participants completed the introductory session, the 1-week app testing period, telephone follow-up, posttest interview, and the satisfaction survey.

Participants used the app in testing for a mean duration of 9 (SD 3, range 7‐16) days. The participant with the longest testing period (16 d) had a delay in follow-up due to illness, which postponed the follow-up interview.

### Demographic Characteristics of Participants

Participants’ mean age was 68 (SD 6) years; 7 were female; 4 were White and 6 were non-White (South Asian, East Asian, or Mixed); and 7 had English as a first language. Participants lived in areas spanning a wide range of neighborhood deprivation (mean IMD decile 4, SD 2, range 1‐8). Mean years of full-time education was 14 (SD 3). Refer to Table 1 for full demographic details.

Most had previous experience with technology (smartphone: n=9, computer: n=8, and tablet: n=6); and 5 stated that they were comfortable or highly comfortable using it.

Common dementia risk factors included hypertension and hearing impairment (n=6 each), physical inactivity and depression (n=4 each), and diabetes (n=3). Moreover, 6 participants had 1‐2 risk factors, and 4 participants had 4 or more.

Among participants enrolled in the 1-week testing—5 chose hypertension, 2 hearing impairment, 2 diabetes, and 1 depression.

**Table 1.** Demographic characteristics of participants (n=10).

Characteristic	Value
Age (y), mean (SD; range)	68 (6; 60‐77)
Years of full-time education, mean (SD; range)	14 (3; 10‐18)
Index of Multiple Deprivation Decile (IMD)^a^, mean (SD; range)	4 (2; 1-8)
Sex, n	
Male	3
Female	7
Ethnicity, n	
White British	4
White Irish	2
South Asian (Pakistani, Indian, Bangladeshi)	2
East Asian (Chinese)	1
Mixed (White & Black)	1
Highest education qualification, n	
Lower secondary qualification (CSE/GCSE)	1
Higher secondary qualification (A-Levels/BTech)	6
Bachelor’s degree	3
English as first language	7
Technology use^b^, n	
Used a tablet before	6
Used a smartphone before	9
Used a computer before	8
Owned a tablet with internet access	5
Comfort using technology, n	
Very uncomfortable	0
Somewhat uncomfortable	3
Neutral	2
Somewhat comfortable	4
Very comfortable	1
Dementia risk factors^c^, n	
Less education (<8 y full-time education)	0
Diabetes	3
Hypertension	6
Obesity	2
Smoking	1
Excessive alcohol	1
Physically inactive	4
Socially isolated	1
Subjective hearing impairment	6
Depression	4
Number of dementia risk factors, n	
1	3
2	3
3	0
4	3
5	1

a The IMD decile (1=most deprived, 10=least deprived) is derived from participant postcodes.

b Dichotomous items; only “Yes” responses are shown.

c Participants may have multiple risk factors; n do not sum to 10.

### Quantitative Findings

#### App Use (Minimum and Additional App Use)

All 10 participants completed and exceeded the minimum required tasks during the first week: watching at least 1 video, answering a check-in question, and playing at least 3 games once. Moreover, 5 participants watched the risk factor video more than once, and 7 participants used the in-app messaging (Table 2). Of the 3 participants who did not use the in-app messaging, 1 reported he did not need it, and 2 said they were unaware of the feature although they had learned how to use this feature as part of the introductory coaching session.

Of the 5 participants who had chosen the hypertension module, which includes a feature allowing for daily blood pressure logging, entries were made on average 5 out of 7 days during the study period, and all participants entered it at least 4 times.

All participants exceeded the minimum required level of gameplay activity. Table 3 presents gameplay data, showing mean plays per game of approximately 5‐10 times per week, with Caterpillar Chase most frequently played and Mushroom Match least. Worm Hunter was tested by only 1 participant, who played it 6 times during the week.

**Table 2.** Additional app use (videos, in-app messaging, and check-in).

Characteristic	Value
Videos rewatch frequency, n	
0	5
1	3
2	1
>3	1
Use of in-app messaging^a^, n	
Yes	7
No	3
Average weekly check-in frequency (days)**,** mean (SD; range)^b^	5 (1; 4–7)

a Participants were coded as having used in-app messaging if they initiated or responded to at least one message with the coach. Those with no interaction were coded as non-users*.*

b *Based on data from 5 participants who are on the hypertension module. Average weekly check-in frequency = (total check-in days ÷ total days of app use)×7*.

**Table 3.** Weekly gameplay frequency for each game among participants (n=10).

Game	Participants assigned^a^, n	Plays per week per participant, mean (SD)	Range
Caterpillar Chase	6	10 (5)	6‐18
Hive Finder	4	7 (3)	4‐9
What Word	5	7 (3)	3‐10
Stump Shuffle	6	7 (2)	3‐9
Night Sorter	4	6 (6)	2‐15
Mushroom Match	4	5 (2)	3‐6
Worm Hunter^b^	1	6 (6)	6‐6

a The number of participants assigned to each game varies because the app randomly allocated three games to each participant per week.

b Data for Worm Hunter should be interpreted with caution as only one participant was assigned this game.

#### Posttest App Satisfaction Survey

**Figure 3.**
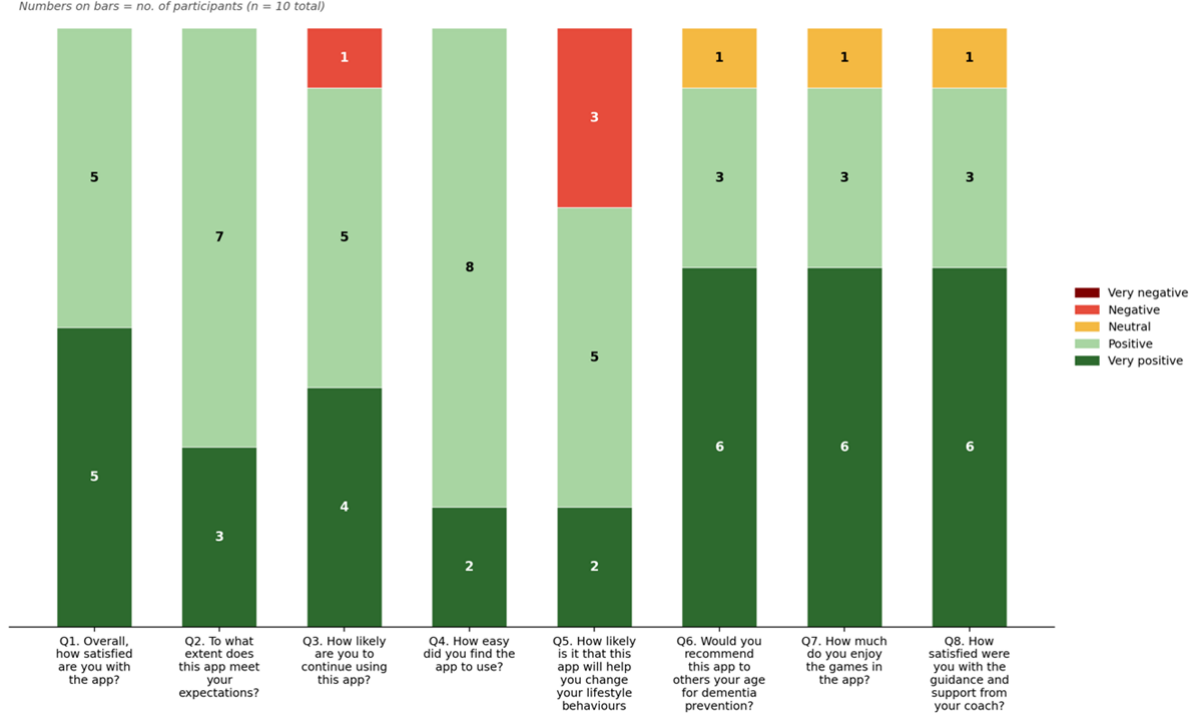
User rating of the app.

Figure 3 presents posttest survey results summarizing participants’ ratings of the app. Response options differed across survey items (eg, satisfaction, likelihood, ease of use, and enjoyment); however, for visualization purposes, responses were harmonized into a standardized 5-point scale ranging from *very negative* to *very positive* based on semantic equivalence. Full survey questions and response labels are provided in Multimedia Appendix 4.

Using the original item-specific response options, all participants reported being either *satisfied* or *very satisfied* with the app and rated it as *easy* or *very easy* to use. All participants felt the app met or exceeded their expectations. Moreover, 9 participants indicated they were *likely* or *very likely* to continue using the app and to recommend it to others their age for dementia prevention. Similarly, 9 participants reported that they *liked* or *liked a lot* the games, and the same number were *satisfied* or *very satisfied* with support from their coach. Regarding the app’s potential to support lifestyle behavior change, 7 participants reported that it was *likely* or *very likely* to help, while 3 reported it was *unlikely*.

No adverse events were reported to the coach or research team during the testing period. However, in posttest interviews, some participants described visual discomfort in relation to rapidly moving or flashing visuals in specific games, which are further described in the qualitative analysis result.

### Qualitative Findings

#### Overview

Guided by the COM-B model, we identified 6 themes, which are detailed in the further sections. Quotes have age ranges to reduce participant identifiability. The most illustrative quotations are presented in the main text, with additional supporting quotations provided in Multimedia Appendix 5.

#### Theme 1: Developing Psychological Capability Through Trial-and-Error App Learning

Many participants initially felt uncertain about the unfamiliar interface but gradually gained confidence through trial-and-error app learning. One participant described figuring out *Night Sorter* without initial instructions:

*I found it [the instructions] a little bit unclear… because it didn’t explain what you were trying to do. I had to spend some effort figuring it out myself. But I got a hang of it at last.*

[P1, age group 60-69 y, White]

Some app features hindered learning due to competing visual cues. One participant described being confused by the green hand icon in Stump Shuffle until the coach clarified:

*Because the “hand” is just so overpowering. It was a great big green hand. It’s telling you, “Press here.” But you’re not pressing there—you should be focusing on the odd logo (the target logo; see Multimedia Appendix 6). I didn’t get that… I didn’t get that at the beginning, but [the coach] explained.*

[P4, age group 60-69 y, Asian ethnicity]

App learning also varied depending on participants’ previous experience with similar technologies. Familiar interfaces made learning more intuitive. When app visuals mirrored interfaces that participants already knew—such as messaging apps.

*Yes, for me and I guess most people, the message one [in-app messaging’s interface] is intuitive, isn’t it? It is like WhatsApp. I thought it pop up the keyboard and everything. That’s good*.

[P1, age group 60-69 y, White ethnicity]

#### Theme 2: Drivers of Motivation—Enjoyment and Personal Goal

This theme describes participants’ motivation to engage with the ENHANCE app, driven by enjoyment of game challenges, satisfaction from receiving virtual rewards, and desire for self-improvement.

##### Enjoyment of Game Challenges Boosts Motivation

Many participants described the games as “fun,” “exciting,” and even said they “got hooked.” They especially appreciated games that were mentally stimulating, with just the right level of challenge—difficult enough to engage their thinking, but not so hard as to be discouraging.

*I think [Hive Finder] is at the right level of challenge because it was pretty challenging for me – I didn’t always get it. I’d run out of time sometimes. But it was fun.*

[P3, age group 60-69 y, Mixed ethnicity]

Conversely, games perceived as too simple led to disengagement:

*I don’t like this [Night Sorter]. Bosh, bosh, bosh. It’s very straightforward. But no, it’s not much fun, is it? It’s a bit stupid, really, isn’t it? Not much thinking needed.*

[P1, age group 60-69 y, White]

Participants also valued the adaptive design of some games, where difficulty adjusted based on performance, helping maintain motivation.

*If you got it wrong, the game [What Word] would give you an easier word to get you more motivated. I like this… Because if it gave me harder ones right after a miss, I’d probably stop and say, “Forget it.”*

[P3, age group 60-69 y, Mixed ethnicity]

##### Desire for Self-Improvement Drives Use

Beyond enjoyment, many participants were motivated by a desire to improve their cognitive skills or overall performance, believing that achieving meaningful personal benefits would drive long-term app use.

One participant described the games as a helpful mental exercise:

*I like games. It’s good for me to do it because that’s not my strong subject anyway. I regard it as just a good exercise…as an activity which is helpful because it really focuses me on my concentration. So I continued.*

[P7, age group 70-79 y, White]

Participants also suggested adding progress visualization features, such as performance trends, believing this would help reinforce personal goals of improving memory and strengthen motivation.

*Is there anywhere that accumulates those scores to give you, say, a weekly, to show you a weekly, ups and downs or to see whether you’ve got better during the week or the month? To give you more incentive to keep trying to beat your (game) score.*

[P8, age group 70-79 y, White]

Similar progress tracking suggestions extended to other features such as the blood pressure check-in (Multimedia Appendix 5).

##### In-App Virtual Rewards Reinforce Use

Many participants found the in-game virtual rewards—flowers for completed activities—provided a sense of achievement and encouraged continued participation. One participant described the flower reward as a meaningful achievement:

*This is a clever idea Yeah. It’s accomplishment to me.*

[P7, age group 70-79 y, White]

However, when rewards failed to appear due to a bug during testing, motivation declined, leading to frustration and disappointment:

*Sometimes if I played the game twice in a session, it didn’t seem to add, even though it says play for one star, and it didn’t, the second time you played it, didn’t seem to add the reward to your watering can…. …This is disappointing. What’s the point then?*

[P2, age group 60-69 y, White]

### Theme 3: Physical Capability Barriers—When Design Overlooks Mobility and Sensory Needs

Some participants with physical or sensory limitations found that certain app features in ENHANCE did not sufficiently support their physical capabilities, creating barriers to engagement. For example, a swipe-based game (*Night Sorter*) was difficult for a participant with reduced movement due to an arm injury. The speed required to play the game interfered with their ability to participate:

*I’ve got a problem in my arm because I’ve had an accident so I can’t do fast for this (Night sorter) The swipe thing is kind of like difficult.*

[P9, age group 70-79 y, Asian]

Concerns were also raised regarding low-contrast visuals and time constraints (Multimedia Appendix 5).

### Theme 4: Discomfort in Gameplay Undermined Motivation

Some participants raised concerns about potential effects from certain games, particularly related to visual discomfort. These experiences undermined motivation and, in some cases, led to disengagement from the games. Some participants reported visual discomfort from specific game elements. For example, *Stump Shuffle*—which involves rapidly moving visuals—was visually demanding:

*When you had a lot of stumps, and they were moving around… Not just one stump was moving, several of them were moving. I find that…visually quite difficult with flashing numbers, flashing letters, that thing… especially for Epilepsy. That might set you off a bit …*

[P2, age group 60-69 y, White]

Similar concerns were raised about Hive Finder due to small, cluttered screen icons (Multimedia Appendix 5).

### Theme 5: Enabling Opportunity on App Use—The Role of Coaching and Hands-On Learning

Participants consistently described initial face-to-face coaching as essential, providing real-time support and reassurance.

*I think it [the introductory session] is very necessary…We can stick to exactly what the coach has shown us. … to have that face-to-face setting up and everything, really.*

[P2, age group 60-69 y, White]

Hands-on use during coaching sessions enhanced opportunity to learn by reinforcing understanding. Several participants suggested encouraging users to perform tasks themselves during coaching sessions.

*[What can be improved in the session?] Yeah, if the coach showed them how to do it once and then had them do it themselves once.*

[P3, age group 60-69 y, Mixed ethnicity]

Participants also appreciated ongoing support through phone calls and in-app messaging after the initial session. This helped maintain engagement by providing a sense of personal care and reassurance.

*I like it [follow-up calls] because you still feel like, oh, somebody really cares.*

[P10, age group 60-69 y, White]

### Theme 6: Boosting Motivation for Behavior Change: Trusted Experts and Personal Stories

Most participants appreciated the ENHANCE app’s educational videos, which combined expert-delivered information with relatable personal stories. This blend of trusted information and real-life experiences encouraged reflection on health behaviors and motivating change.

*Dr. Sherman [Dr. featured inside the video] is going to be telling the truth because he was going to be real life doctor. It’s not like YouTube people… It’s going to be true. I was ready and willing to listen carefully and attend.*

[P4, age group 60-69 y, Asian]

Practical, actionable advice from professionals also motivated participants to apply recommendations in their daily lives. One participant using the diabetes module appreciated the clear dietary guidance:

*The video shows me what to do. That’s very important…certain foods make the blood sugar go quickly up. If we eat potatoes, they’ll make it go up. Glycemic, they call it… That would be really useful and give good tips how to improve it.*

[P9, age group 70-79 y, Asian]

Personal stories created emotional resonance that deepened engagement and relevance. One participant expressed a clear preference for human stories over diagrams:

*I like that it has someone’s personal story because it makes it more relatable. personally, I like to see something. I relate more to seeing that human rather than diagrams.*

[P1, age group 60-69 y, White]

Furthermore, a participant living with long-term depression found comfort in the simplicity of a story from the “Better Mood” module (Multimedia Appendix 5)

Table 4 summarizes the themes mapped to the COM-B model and their associated design implications, discussed further in the Discussion section.

**Table 4.** Design Implications.

COM-B^a^ domain and relevant themes	Design implications
Capability	
Theme 1. Developing capability through trial-and-error learning	Design interfaces that allow users to try, make mistakes, and learn without penalties Use familiar interface elements and game formats (eg, WhatsApp-style chat, card matching, Sudoku, word games) to support intuitive learning.
Theme 3. When design overlooks mobility and sensory needs	Use large fonts and high color contrast Avoid game features that require fine motor skills or fast reactions.
Opportunity	
Theme 5. The role of coaching and hands-on learning	In-person human coaching is essential to support effective app use. Introductory sessions should prioritize experiential learning over didactic instruction. Telephone follow-up coaching is generally preferred over in-app chat, though chat should remain available as an option Ensure access to a device and reliable internet connectivity. Family involvement can be beneficial and should remain an optional form of support.
Motivation	
Theme 2. Enjoyment, personal goals, and in-app rewards increase motivation	Incorporate fun games to boost overall app engagement. Design games with light problem-solving and adaptive difficulty Keep games short and update game content regularly Use consistent virtual rewards linked to personal goals (eg, better brain health) Use simple, easy-to-understand visual progress tracking linked to users’ personal goals.
Theme 4. Visual discomfort in gameplay undermines motivation	Avoid flashing visuals, rapid motion, or dense layouts. Offer visually calm alternatives or adjustable display settings. Test games with users who have sensory sensitivities.
Theme 6. Trusted experts and personal stories increase motivation for behavior change	Combine trusted expert input with relatable peer narratives Use clear, actionable health advice Present information in simple multimedia formats to support reflection and behavior change.

a COM-B: capability, opportunity, motivation – behavior.

## Discussion

### Principal Findings

Our 1-week usability testing suggests that the ENHANCE intervention prototype is acceptable and usable for older adults. All participants met minimum-use targets, with many engaging further, making extensive use of core features (games, videos, and questionnaires). Most also used the in-app messaging feature. All reported high satisfaction with the app and coaching sessions on the postuse survey.

Qualitative interviews identified several facilitators of engagement, such as (1) face-to-face coaching support, (2) appropriately challenging and adaptive games, (3) in-app virtual rewards, (4) visual progress tracking, (5) familiar interface elements, and (6) health information combining expert advice with personal stories. Barriers included unclear interfaces or instructions, design that did not adequately account for users’ physical limitations, and visual discomfort linked to specific aspects of game design.

### Comparison With Previous Work

Overall, our positive usability and acceptability findings are consistent with the Prevention of Dementia Using Mobile Phone Applications (PRODEMOS) study—the only other UK-based, app-based dementia prevention program targeting underserved populations. The PRODEMOS reported that among its UK participants (n=600), 69% found the app acceptable, 71% considered it feasible, and 77% viewed it as appropriate at 3-month follow-up [36].

While both our study and PRODEMOS included features such as SMART goal setting, educational content, cognitive games, and health tracking, ENHANCE offered more intensive human interaction—including face-to-face onboarding, weekly telephone follow-ups, and in-app messaging with a coach. In contrast, PRODEMOS delivered initial sessions remotely due to COVID-19 restrictions, with follow-up support limited to asynchronous in-app chat. Participants in PRODEMOS reported difficulties staying engaged, partly attributing this to delays in coach responses [36,37]—a challenge similarly noted in Healthy Ageing Through Internet Counselling in the Elderly (HATICE) internet-based dementia prevention trial [38,39]. .

Our qualitative findings highlighted the value of in-person coaching. Participants described face-to-face onboarding as “necessary” for real-time support and reassurance—echoing findings from the HATICE study [38]. For ongoing support, telephone check-ins were more acceptable than in-app chat: all participants completed phone calls, while only 7 out of 10 used the chat feature. Nonusers tended to be older, less educated, and less familiar with tablets, and were unaware of the chat function despite onboarding demonstrations. This aligns with evidence that human-led approaches—in-person or voice-based coaching—are preferred by older adults and may foster deeper engagement than text-based or AI-driven approaches, such as chatbots [40,41]. In-app chat also posed greater challenges for those with lower digital literacy consistent with evidence that text-based interfaces can present direct barriers when reading or digital skills are limited [42].

While PRODEMOS assessed usability at 3 months, ENHANCE was evaluated after just 1 week, leaving its long-term engagement and feasibility untested. PRODEMOS reported a decline in task completion from 91% in the first 2 weeks to 41% by month 3 [36], reflecting a common pattern of reduced app engagement over time due to loss of novelty and motivation [43,44].

### Interpreting Findings Through the COM-B Framework

Using the COM-B framework [32], our qualitative findings can reflect how capability, opportunity, and motivation shaped participants’ engagement with the ENHANCE app.

#### Building Users’ Capability Through Effective App Learning

Our findings indicate that trial-and-error learning fostered app engagement by helping users build confidence and skills in using the app (capability). A key facilitator was the use of familiar interface elements—design features similar to those older users already know—which aligns with evidence that recognizable visuals, layouts, and game mechanics reduce cognitive load and enhance usability, particularly for those with limited digital experience [45-48]. In the UK context, games like Scrabble and matching-pair card games are widely recognized by older adults, and several of our games adopted similar mechanics, appearing to lower the cognitive barrier to entry and support a smoother learning curve.

Additionally, we found some app features exceeded users’ physical capabilities, reinforcing the need to design in alignment with older adults’ motor and visual abilities. Games requiring fine motor skills or fast reactions should be avoided, and interfaces should prioritize large fonts, clear instructions, and limited simultaneous visual cues—consistent with existing recommendations for age-inclusive design that accommodate age-related changes in motor and cognitive function [45-48].

#### Creating Opportunity Through Coaching

Our study highlights human coaching as a critical factor supporting app engagement, aligning with evidence on blended approaches that combine digital tools with human support, particularly for socioeconomically disadvantaged populations [49]. Personalized coaching can help overcome low digital literacy and complex life challenges (eg, financial barriers to healthy food and poor living conditions) by discussing practical workarounds during sessions [50]. Participants also consistently preferred hands-on, experiential learning during coaching sessions over passive instruction. This supports findings showing that trial-and-error—or “generate-and-correct”—learning enhances recall more effectively than passive study-only approaches [51]. These insights highlight the value of coaching models that emphasize user-led exploration and problem-solving.

#### Other Opportunities-Related Factors

Our findings partially align with previous research identifying device access, time availability, and family involvement as key opportunity-related factors in app engagement [21,52]. In our study, device and internet barriers were mitigated by providing tablets and connectivity. Time availability, however, did not emerge as a major constraint—possibly reflecting participants’ intrinsic motivation or the role of financial incentives in this study.

Although family involvement is often cited as a facilitator [21,34,52], it was notably absent in our findings: despite being asked during onboarding whether they wanted family support, no participant involved family during the testing period, commonly due to living alone or lacking tech-savvy relatives nearby. For older adults from lower socioeconomic or ethnic minority backgrounds, where geographic distance and limited digital literacy within families are common, family support may not be a practical resource [49]. External coaching may therefore serve as a more feasible alternative, with family involvement supplementary where available.

#### Strengthening Motivation Through App Design

Several app features enhanced both automatic (eg, positive emotional responses) and reflective motivation. These included the games, the combination of professional advice and peer narrative in health information delivery, the desire to improve cognition, and visual feedback and in-app virtual rewards.

#### The Use of Games

Games were found to be a major source of engagement. Participants enjoyed the cognitive challenge, especially when difficulty was appropriately balanced and adapted to their performance—experiences resembling flow, a state of focused immersion to enhance intrinsic motivation and long-term engagement [53,54]. A meta-analysis found that gamified cognitive training yields greater motivation and engagement than less gamified alternatives [55].

Several design choices likely contributed—games focused on light problem-solving tasks with adaptive difficulty; provided only positive feedback with no penalties, were brief (under 8 min per session); and introduced new games weekly—all consistent with recommendations for apps or games targeting older adults [46,47]. Competitive elements, such as leaderboards or public performance displays, were deliberately excluded, in line with evidence that these can deter older users [46].

#### The Use of Expert Guidance and Peer Stories to Deliver Health Information

Our findings suggest that health information was best received when combining expert guidance with peer stories. While older adults tend to trust health care professionals over social media or family and friends [56,57], they also find peer narratives relatable and motivating. Other studies support the value of narrative health communication: personal stories are more emotionally engaging than fact-based messages and are particularly effective at influencing behavioral intentions [58,59]

#### The Use of Visual Progress Tracking and In-App Virtual Rewards

Participants suggested that visual progress tracking (charts showing improvement in games or behaviors) and in-app virtual rewards (flowers) may support user engagement and health behavior change. Evidence from physical activity apps reinforces this: visual feedback and virtual rewards were the most effective features for predicting actual activity, outperforming elements like leaderboards [60], and rewarding users with virtual currency earned through steps significantly increased activity over 6 months, particularly among sedentary and high-BMI individuals [61]. However, overly complex visualizations of progress tracking can be unclear to older users [62], and extrinsic rewards may undermine long-term motivation once an internally rewarding engagement loop is established [63]. Feedback and rewards should therefore be simple, easy to understand, and aligned with users’ personal goals to support long-term motivation.

### Strengths and Limitations

This study has several strengths. Recruitment via community settings serving underrepresented groups yielded a sample spanning ethnic minority backgrounds and a wide range of neighborhood deprivation, addressing a key gap in usability evidence for inclusive digital prevention [11]. We used multiple data sources—questionnaires, interviews, and system logs—consistent with the Medical Research Council’s guidelines on mixed methods [64] and evidence that combined approaches provide a more comprehensive usability picture than single methods alone [65]. The 1-week testing period captured how user experience evolved across repeated use rather than relying on a single interaction [66], and participants used the app in their natural settings, supporting ecological validity.

Limitations include the 1-week timeframe, which leaves longer-term usability untested, and the small, London-based sample, which limits generalizability to rural areas and other countries. Social desirability bias may have affected interviews, although backend usage data showing use beyond minimum requirements suggests genuine engagement. Despite targeting underserved settings, the sample had relatively high educational attainment; usability barriers may be greater among those with lower literacy. High engagement occurred under supported conditions—device provision, face-to-face onboarding, coach support, and study compensation—which may not reflect real-world implementation. Finally, eligibility relied on not having self-reported dementia without formal clinical assessment. Future studies should incorporate more rigorous eligibility measures.

### Conclusion

This study explored how community-recruited older adults, including those underrepresented in dementia prevention trials, engaged with the ENHANCE app over 1 week. The intervention was well-received, with high engagement, positive feedback, and strong usability and acceptability across participants. Key facilitators included face-to-face coaching, familiar interface design, appropriately challenging games, visual progress tracking, and health content that combined expert advice with peer stories. These findings offer practical design insights for developing inclusive digital dementia prevention tools that better accommodate diverse older adults, while future research is needed to evaluate maintained long-term engagement.

## Supplementary material

10.2196/92800 Multimedia Appendix 1 Telephone screening questions.

10.2196/92800 Multimedia Appendix 2 Sociodemographic and Risk Factor Questionnaire.

10.2196/92800 Multimedia Appendix 3 Guideline for posttest feedback interview.

10.2196/92800 Multimedia Appendix 4 Posttest survey.

10.2196/92800 Multimedia Appendix 5 Supporting quotes.

10.2196/92800 Multimedia Appendix 6 Screenshots of Stump Shuffle.

## Abbreviations

ACTIVE: Advanced Cognitive Training for Independent and Vital Elderly
COM-B: capability, opportunity, motivation—behavior
CSQ: Client Satisfaction Questionnaire
ENHANCE: Tailored Intervention for Brain Health and Cognitive Enrichment
FINGER: Finnish Geriatric Intervention Study to Prevent Cognitive Impairment and Disability
HATICE: Healthy Ageing Through Internet Counselling in the Elderly
IMD: Index of Multiple Deprivation
PHQ-2: Patient Health Questionnaire−2
PRODEMOS: Prevention of Dementia Using Mobile Phone Applications
SMART: specific, measurable, achievable, relevant, time-bound
