# Supplementary material for: Usability of the Coach-Supported Dementia Prevention App ENHANCE (Tailored Intervention for Brain Health and Cognitive Enrichment) in Older Adults: 1-Week Mixed Methods Study
Source: JMIR Aging. 2026 Jul 23;9:e92800. doi: 10.2196/92800 (PMC13395424; doi:10.2196/92800)
Supplement: Multimedia Appendix 1 [file aging-v9-e92800-s001.docx]

**Telephone screening questions**

| **No.** | **Question** |  |
| --- | --- | --- |
| 1 | Age: ________________years | |
| 2 | Do you have untreated diabetes? | ☐ Yes  ☐ No |
| 3 | Do you have untreated hypertension (high blood pressure)? | ☐ Yes  ☐ No |
| 4 | Have you been told that your BMI (Body Mass Index) is over 30, which is considered obese for your height and weight? | ☐ Yes  ☐ No |
| 5 | Do you smoke? | ☐ Yes  ☐ No |
| 6 | Do you consume more than 21 units of alcohol per week? (Please refer to the guidance) | ☐ Yes  ☐ No |
| 7 | Do you exercise (including walking) for more than 2.5 hours per week? | ☐ Yes  ☐ No |
| 8 | During the last month, have you often been feeling down, depressed, or hopeless?* | ☐ Yes  ☐ No |
| 9 | During the last month, have you often been bothered by having little interest or pleasure in doing things?* | ☐ Yes  ☐ No |
| 10 | In a typical month, how often did you chat or do something enjoyable with friends or family? (Including people you live with) **#** | ________ times per month |
| 11 | Do you have any difficulty hearing (for example, when visiting family or friends, or hearing the TV at a volume that is comfortable for others)? | ☐ Yes  ☐ No |
| 12 | How many years of full-time education do you have? **^** | ______(years) |
| 13 | Have you ever been told by a doctor that you have dementia? | ☐ Yes  ☐ No |
| 14 | Do you have any physical or mental disabilities that you believe may significantly impact your ability to use mobile phones, tablets, or computers? | □ Yes (Please specify: _________)  □ No |
| **Notes for researchers (This section should NOT be shown to participants):**  *** If both Q8 & Q9 were answered 'Yes,' the participants were classified as 'depressed,'**  **# For Q10 : If participants answered less than 4 times per month, they were classified as 'socially isolated.'**  **^ For Q12: Participants with less than 8 years of full-time education were classified as having 'less education’.** | | |

**
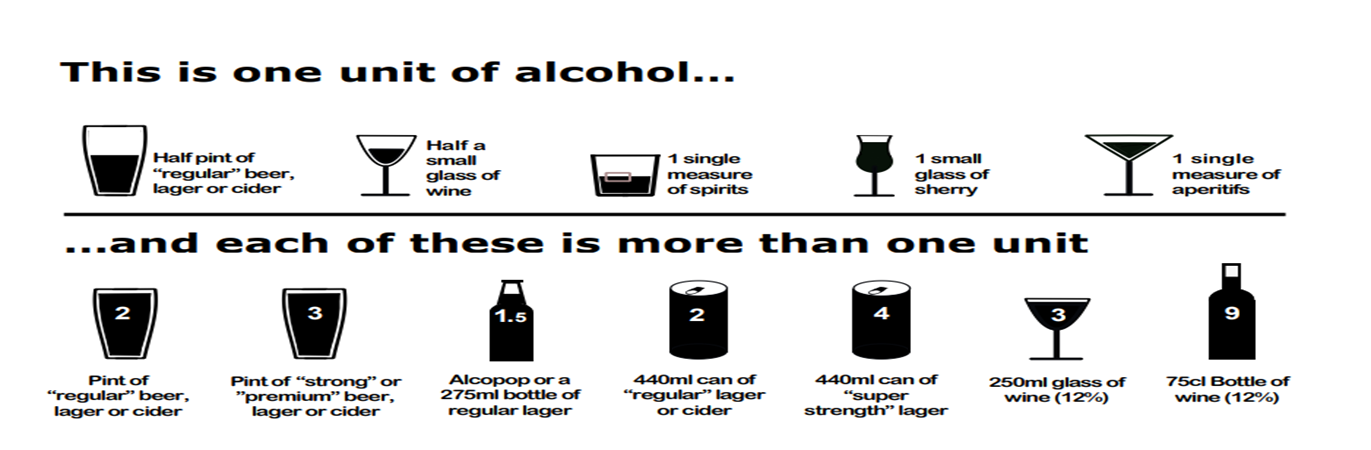
**
