# Supplementary material for: Usability of the Coach-Supported Dementia Prevention App ENHANCE (Tailored Intervention for Brain Health and Cognitive Enrichment) in Older Adults: 1-Week Mixed Methods Study
Source: JMIR Aging. 2026 Jul 23;9:e92800. doi: 10.2196/92800 (PMC13395424; doi:10.2196/92800)

**Socio-demographic and Risk Factor Questionnaire**

**Instruction to researcher/coach: Please complete this questionnaire together with the participant. It takes approximately 15 minutes. This questionnaire collects demographic information, serves a screening purpose, and assists the coach in understanding the participant's dementia risk profile, guiding them to select relevant risk factors within the ENHANCE app. If screening has already been completed for the participant (Appendix 1) and they have been confirmed as eligible, please skip Q5 and Q7–16, and use the existing screening results to review the participant's risk profile.**

| Please indicate the information below by putting your answer or by a putting a 🗸 in the □. | | | |
| --- | --- | --- | --- |
| **1.** | **Your age:** |  | |
| **2.** | **Sex** | □ Male  □ Female  □ Other | |
| **3.** | **Your ethnicity:** | □ Whites | |
|  |  |  | □ White-British  □ White-Irish  □ Whites-Any other background (Please specify: _____________________) |
|  |  | □ Asian or Asian British | |
|  |  |  | □ Pakistani  □ Indian  □ Bangladeshi  □ Chinese  □ Any other Asian background (Please specify: _____________________) |
|  |  | □ Black or Black British | |
|  |  |  | □ Caribbean  □ African  □ Any other black background (Please specify: _____________________) |
|  |  | □ Mixed ethnicity | |
|  |  |  | □ White and Asian  □ White and Black Caribbean/African  □ other mixed background (Please specify: _____________________) |
|  |  | □ Any other ethnicity: ___________ | |
| **4.** | **Your Postal code** |  | |
| **5.** | **How many years of full-time education do you have? ^** | _____________years  What is your highest education qualification?  ______________________ | |
| **6.** | **What is your occupation?** | (Please tick one box only)  -Managerial / professional □  -Never worked / long term unemployed □  -Routine and manual □  -Sick / disabled and unable to work □  -Retired □  -Other □  -Home carer □ | |
| **7.** | **Do you have untreated hypertension (high blood pressure)?** | □ Yes □ No | |
| **8.** | **Do you have untreated Diabetes Mellitus?** | □ Yes □ No | |
| **9.** | **Have you been told that your BMI (Body Mass Index) is over 30, which is considered obese for your height and weight?** | □ Yes □ No | |
| **10.** | **Do you smoke regularly?** | □ Yes □ No | |
| **11.** | **Do you consume more than 21 units of alcohol per week? (Please refer to the guidance at the end of the document)** | □ Yes □ No | |
| **12.** | **Do you exercise (including walking) for more than 2.5 hours per week? (See reference picture below)** | □ Yes □ No | |
| **13.** | **During the last month, have you often been feeling down, depressed, or hopeless? *** | □ Yes □ No | |
| **14.** | **During the last month, have you often been bothered by having little interest or pleasure in doing things? *** | □ Yes □ No | |
| **15.** | **In a typical month, how often did you chat or do something enjoyable with friends or family? (Including people you live with) #** | □ Yes □ No | |
| **16.** | **Do you have any difficulty hearing (for example, when visiting family or friends, or hearing the TV at a volume that is comfortable for others)?** | □ Yes □ No | |
| **17.** | **How comfortable are you using technology?** | □ Very uncomfortable  □ Somewhat uncomfortable  □ Neutral  □ Somewhat comfortable  □ Very comfortable | |
| **18.** | **Do you have any physical or mental disabilities that you believe may significantly impact your ability to use mobile phones, tablets, or computers?** | □ Yes (Please specify: ____________________________)  □ No | |
| **Notes for researchers (This section should NOT be shown to participants):**  *** If both Q13 & Q14 were answered 'Yes,' the participants were classified as 'depressed'.**  **# For Q15: If participants answered less than 4 times per month, they were classified as 'socially isolated.'**  **^ For Q5: Participants with less than 8 years of full-time education were classified as having 'less education’.** | | | |


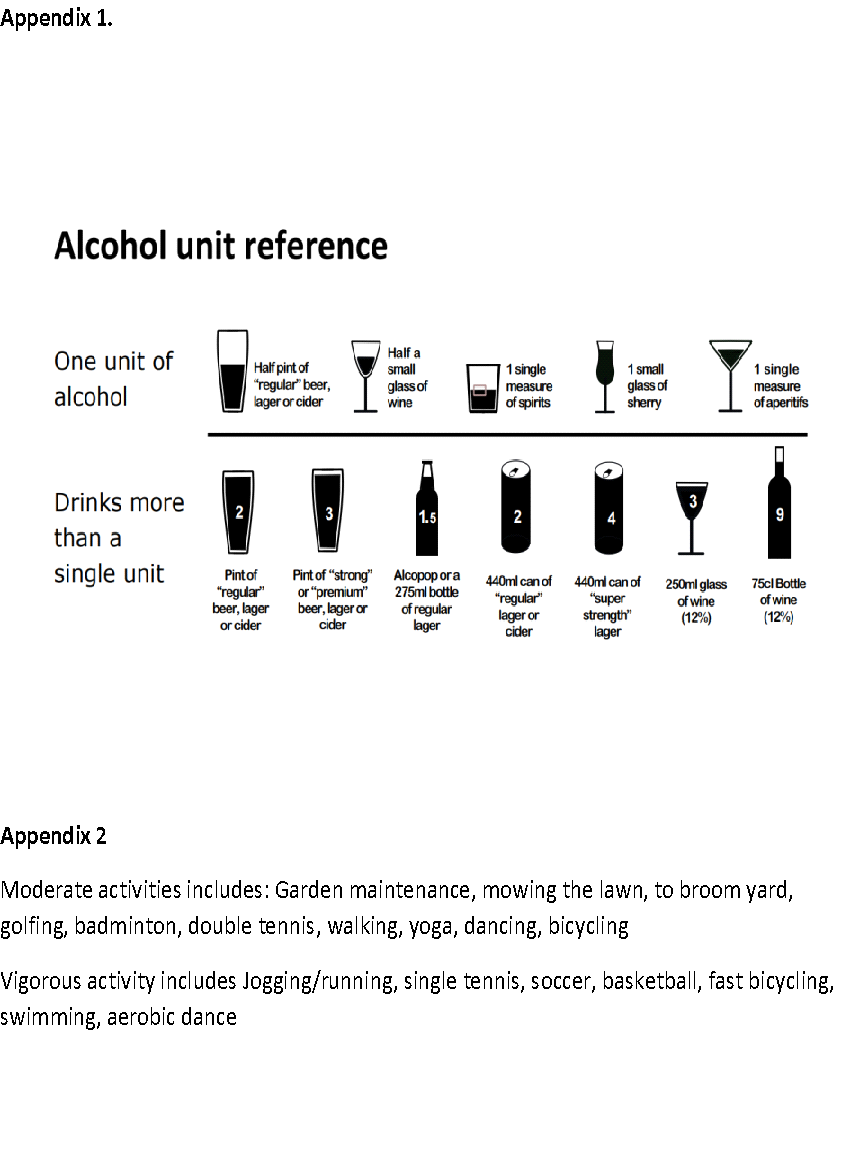

Supplement: Multimedia Appendix 2 [file aging-v9-e92800-s002.docx]
