# Supplementary material for: Usability of the Coach-Supported Dementia Prevention App ENHANCE (Tailored Intervention for Brain Health and Cognitive Enrichment) in Older Adults: 1-Week Mixed Methods Study
Source: JMIR Aging. 2026 Jul 23;9:e92800. doi: 10.2196/92800 (PMC13395424; doi:10.2196/92800)
Supplement: Multimedia Appendix 3 [file aging-v9-e92800-s003.docx]

**Guideline for post-test feedback interview**

- The interview will last approximately one hour.
- The researcher will conduct the interview face to face.
- This is a semi-structured debriefing interview. That means this allows interviewers the flexibility to elaborate on questions, move to another section if the participants talk about it, or skip those questions that may not be relevant to the participant.

**Brief procedure:** After a brief introduction about the interview (Step 1), researchers will ask for participants' general feedback on the app (Step 2), and then a 'think-aloud' for each section of the app (Step 3a-3d). This will include the meadow scene, games, selected risk factor module, and messaging system. Following the think-aloud, researchers will ask participants for feedback on the coaching sessions (Step 4), as well as address any remaining questions not covered earlier. The session will then be closed.

| **Step 1. Provide a short introduction about the interview** | |
| --- | --- |
| **What researchers need to do?** | **Example of script/questions** |
| - Thank the participants, for taking part - Introduce yourself - Purpose and duration of this testing - Introduce think aloud technique - Reminders about audio-recording of the interview and we make notes - Ask if there are any questions | **Intro: *‘****" My name is [Your Name] Thank you so much for using our app over the past week! Today I’ll be asking you a few questions about your experience with the Enhance app. Today’s interview will last about 1 hour. During the session, I’ll ask you to open the enhance app and explore it just like you did over the past week. As you go through it, I’d like you to share* ***your thoughts out loud****. Let me know what you liked, didn’t like, or if you had any struggles. Your frank feedback will help us make the app better for people like you. After that, I’ll ask you some general questions we haven’t covered yet.*  *I want to let you know that* ***I'll be recording our conversation*** *to make sure I don't miss any of your important comments. I’ll also* ***be taking some notes*** *as we go along.*  *Before we start,* ***do you have any questions*** *about the interview process? If not, we can begin right away!’* |
| **Step 2. Interview participants to feedback of the overall app** | |
| **What researchers need to do?** | **Example of script/questions** |
| - Get general feedback about the app | *‘First, I’d like to ask you some general questions about your overall experience and views on the app’*  **Examples of Questions:**  *After using the ENHANCE app this past week, what are your thoughts on it?*   - *Do you think you will continue using the app over the next few months if you have the app? Why or why not? What would make it easier or harder to do so? What changes or support would help you keep using it?* - *Is there anything in the app or questions which you found off-putting, insensitive, patronising or culturally inappropriate?* - *Do you think this app is easy to use for people with hearing or visual impairments, those whose first language isn’t English, people with less education, or those with limited experience using apps? (These questions will only be asked if your participant belong to one of these groups)* - *Did you find the app easy to navigate overall?* - *What did you like most about the app?* - *What did you like least about the app?* - *How do you feel about the app's layout, design, and language?* - *Were there any moments where you felt stuck or confused when you were using the app at home (login, entering the library)? What did you do? What do you think caused the issue?* - *How would you improve the app?* |
| **Step 3a. Ask the participants to open the app and ask for their feedback on the Meadow Scene** | |
| **What researchers need to do?** | **Example of questions** |
| - Ask the participant to explore the Meadow scene and share their feedback. | - *What were your thoughts when you first opened the app and saw the meadow scene? Did you like it or not? What could be improved?* - *Did you know what to do when you first opened the app?* - *Can you click this watering can icon on the left lower corner? Can you tell me what this does? Could you try planting a tulip bulb in the meadow for me?*   ***What to observe?***   - *Where they clicked first when they opened the Meadow scene. Did they know where to click, or did they click in the middle of the scene expecting something to happen?* - *Any navigation challenges they faced when planting a tulip.* - *If they know how to exit the watering can section if needed.* |
| **Step3b. Ask the participants to enter the game’s library and play games** | |
| **What researchers need to do?** | **Example of questions** |
| - Test if the participant knows how to navigate to the game’s library from the Meadow scene. - Ask the participant to play two-three games and share their feedback. | **Starts with….**  *Can you go to the game library and select a game to play with (Starting from the meadow scene)? (Researchers will observe any navigation challenges when they entered the games library)*  **After the participants played the game:**   - *Did you enjoy the game? Why or why not?* - *Was there anything about the games that frustrated you last week?* - *Do you think you would like to continue playing these games in the coming weeks if you have the app? Why or why not? What changes or support would help you keep playing the games?* - *I noticed that you _, can you tell me why?*   **Other questions about the games:**   - *I noticed that you didn’t play any games this week. Would you mind saying why?* - *I noticed you played ___ (name of the game) for a few minutes in the past week. What did you like about the game? And what made you stop playing?* - *We suggested playing the games at least three times a week. Were you able to do that? Was it hard to reach that number? Did you play more? Why or why not?* - *How can the coach help you use the games on the app more?* - *There’s a limit of 10 plays per game (10 tokens) each week. How do you feel about this game play limit?* - *The enhance app has 10 games in total, and you get 3 random ones each week. How do you feel about that? Would you prefer a different way? If so, why?* |
| **Step 3c. Getting feedback about the selected risk factor module (check-in questions, videos)** | |
| **What researchers need to do?** | **Example of questions** |
| - Ask for the feedback on the selected risk factor module (i.e. Check in questions and videos for the first week) | **Starts with…**   - *I noticed you [completed/didn’t complete] the [name of the activity] last week. How was your experience with that (if completed)? Do you mind sharing why you didn’t complete the activities (If didn’t complete)?*   **Check-in questions:**   - *How did you find the check-in questions? Do you like them or not? Why?* - *Did you face any challenges completing the check-in questions? (For example, for hypertension, were you able to measure and enter your blood pressure in the app last week? If not, what challenges did you encounter?)* - *Do you think the check-in questions are easy to answer? (Consider the language, number of options, and how you select answers—like using a slider.)* - *Do you think the check-in questions help you monitor your behaviour?* - *How can these questions be improved?*   **Videos**   - *What did you like or dislike about the video you watched last week?* - *How did you find the content? Was it familiar, or would you prefer different topics?* - *How could the video be improved in terms of content, clarity, speed, or length?* |
| **Step 3d. Getting feedback about the messaging system** | |
| **What researchers need to do?** | **Example of questions** |
| - Test if they know how to message their coach on the app - Ask for the feedback on the messaging system | ***Starts with….***  *‘Do you know how to message the coach via the messaging system in the app? Can you show me how to do it’*  ***Questions on the messaging system:***   - *In the past week, did you message him/her via the app? If so, how was that experience for you?* - *How easy was it to connect with your coach through the app using the messaging system? Is it easy or difficult to navigate or type your message in the app? Can you tell me what it was that you found easy or difficult.* |
| **Step 4: Interview participants about coaching onboarding session & follow up coaching session** | |
| **What researchers need to do?** | **Example of scripts or questions** |
| - End the think-aloud and get their feedback on the coaching sessions (onboarding & follow up coaching session) | *"Thank you for taking the time to explore the app. Now, I would like to ask you some questions about the coaching sessions. You’ve attended two coaching sessions. The first was the in-person onboarding session, where you learned how to set up and use the app and select a risk factor to start with. The second was a remote follow-up session, where the coach reviewed your progress with you. I’d love to get your feedback on both sessions. Shall we start by discussing the onboarding session.?’*  **Questions on onboarding session:**   - *How was your experience with the onboarding session- that is the first coaching session? Anything you liked or disliked? What could be improved?* - *Do you think the onboarding session helped you get familiar with the app and feel comfortable using it?* - *The onboarding session lasts around 45 minutes. How did you feel about the length?* - *Was there anything you felt should have been covered in the onboarding session but wasn’t?* - *Are there any aspects of the onboarding session that you found frustrating or unhelpful?*   **Questions on the remote coaching meeting (the second meeting with the coach):**   - *How was your experience with the 2^nd^ coaching session (The remote one)? Anything you liked or disliked? What could be improved?* - *Do you think the second coaching session helped you use the app better?* - *Do you think the second coaching help motivate you to make changes in your behaviour?* - *The second coaching session lasts around 30 minutes. How did you feel about the length?* - *The second coaching session is a remote session. How did you feel about that?* - *Was there anything you felt should have been covered in the second session but wasn’t?* - *Are there any aspects of the second session that you found frustrating or unhelpful?*   **Overall:**   - *How can the coaching sessions be improved to support long-term use of the app?* - *What did you find most valuable in these sessions?* - *What did you find most unhelpful in these sessions?* - *How effective was the coach in helping you set achievable goals?* - *How effective was the coach in helping you to support your progress?* - *How effective was the coach in helping you to resolve any difficulties you encountered (technical difficulty, motivation related difficulties)?* |
| **Step 5. Ending the debriefing session** | |
| - Thank user for their valuable participation. - Ask user if they have any final thoughts or suggestions about their experience with the app? - If not, researchers end the session and keep the tablet and give £50 voucher to participant. | |
