# Supplementary material for: Usability of the Coach-Supported Dementia Prevention App ENHANCE (Tailored Intervention for Brain Health and Cognitive Enrichment) in Older Adults: 1-Week Mixed Methods Study
Source: JMIR Aging. 2026 Jul 23;9:e92800. doi: 10.2196/92800 (PMC13395424; doi:10.2196/92800)
Supplement: Multimedia Appendix 4 [file aging-v9-e92800-s004.docx]

**Post-test Survey**

| **Post-test Survey**  **Participant code:**  **Date of completion:**  **Name of researcher:** | | |
| --- | --- | --- |
| **Instructions: Please answer the following questions about your one-week experience with the app. Indicate your response by circling the appropriate answers that best reflects your opinion for each question. Your feedback is valuable and will help us improve the app.** | | |
| 1. | Overall, how satisfied are you with the app? | Very dissatisfied (1) Dissatisfied (2) Neutral (3) Satisfied (4) Very satisfied (5) |
| 2. | To what extent does this app meet your expectations? | Far Below my expectation (1) Below my expectation (2) Neutral (3) Meets my expectation(4) Exceeds my expectation (5) |
| 3. | How likely are you to continue using this app (if you still have the app and tablet)? | Very unlikely (1) Unlikely (2) Neutral (3) Likely (4) Very Likely (5) |
| 4. | How easy did you find the app to use? | Very difficult (1) Difficult (2) Neutral (3) Easy (4) Very Easy (5) |
| 5. | How likely is it that this app will help you change your lifestyle behaviours? | Very unlikely (1) Unlikely (2) Neutral (3) Likely (4) Very Likely (5) |
| 6. | Would you recommend this app to others your age for dementia prevention? | Definitely would not (1) Probably would not (2) Not sure (3) Probably would (4) Definitely would (5) |
| 7. | How much do you enjoy the games in the app? | Dislike a lot (1) Dislike (2) Neutral (3) Like (4) Like a lot (5) |
| 8. | How satisfied were you with the guidance and support from your coach? | Very dissatisfied (1) Dissatisfied (2) Neutral (3) Satisfied (4) Very Satisfied (5) |
