# Supplementary material for: Usability of the Coach-Supported Dementia Prevention App ENHANCE (Tailored Intervention for Brain Health and Cognitive Enrichment) in Older Adults: 1-Week Mixed Methods Study
Source: JMIR Aging. 2026 Jul 23;9:e92800. doi: 10.2196/92800 (PMC13395424; doi:10.2196/92800)
Supplement: Multimedia Appendix 5 [file aging-v9-e92800-s005.docx]

| **Supporting quotes** | | |
| --- | --- | --- |
| **Theme** | **Codes** | **Quotes:** |
| **Theme 1 Developing psychological capability through trial-and-error app learning** | **Trial and error learning** | *‘At first, I didn't get it. But then on the second round, I got it. I figured out how to do it…At first, I didn't get that, but then I thought about it, and I thought, OK, it's simple. ‘(P3, age group 60-69y, Mixed Ethnicity)* |
|  | **Confusing visual cues impedes learning** | *‘Then the finger (green hand icon) confused me in the beginning. But after playing a couple of times, I got the hang of it. It's just that it's hiding that and you're trying to figure out which one is odd one out, really.’ (P5, age group 70-79y, White)* |
|  | **Prior experience aiding learning** | ‘*I found [the blood pressure feature] really easy. Yes, there was no difficulty. It was obvious because it matches my blood pressure monitor*.’ (P2, *age group 60-69y*, White) |
| **Theme 2. Drivers of motivation: enjoyment and personal goal** | **Desire for self-improvement drives use** | *‘it's fact that draws you back onto it (losing the game- worm Hunter) that you want to get better… it does make you want to go back and try and beat …. (P5, Age group 70-79, White)* |
|  | **Progress tracking motivates self-improvement and app use** | *“Like a progress chart (for the games)—yes, a little chart or something—to show how you’ve been doing. Because for me personally, it’s like, ‘Oh, I want to beat that score and see how I did.’” (P10, age group 60-69y, White).* |
|  | **Progress tracking motivates self-improvement and app use** | *: “Same for the blood pressure monitor… I did it each day, but it didn't show you, again, what happened the previous time or over the week. I think for blood pressure, especially, that would have been good, .see if there is improvement.” (P2, age group 60-69y, White)* |
|  | **In-app virtual rewards reinforce use** | *‘I like the tulips. Any flowers are nice. I'm looking forward to decorating this (meadow) it adds cumulatively, presumably each day, each time you play it.’ (P2, age group 60-69y, White)* |
| **Theme 3: Physical capability barriers: When design overlooks mobility and sensory needs** | **Colour contrast** | *“If you make the boulders a bit darker colour, not just grey, because it's too near the beige colour, because it is difficult to see....and you haven't got much time already in the game. the stone itself, black or something.” (P7, age group 70-79y, White)* |
| **Theme 4: Discomfort in gameplay undermined motivation** | Dizziness | "I find that the pictures. You see they're small (icons on the Hive). They're small and then my eyesight is no good. Unable to play. I won't play Hive (Finder), because it makes me feel very dizzy…" P6 (60-69y, Asian) |
| **Theme 5. Enabling opportunity on app use: The role of coaching and hands-on learning** | **Face-to-face coaching** | “You can ask questions, and they [the coach] can see what you’re doing. You’re both looking at the same page, basically—when the questions arise.” (P 7, age group 70-79y, White) |
|  | **Hands-on use during coaching** | "Yeah, if the coach showed them how to do it once and then had them do it themselves once." (P3; 60-69y, Mixed) |
|  | **Hands-on use during coaching** | "Well, I suppose saying, 'Can you show me what I've just told you?'... Demonstrate that you can do what you said you can do." 9P1;60-69y, White) |
|  | **Importance of follow-up coaching session** | Yeah, because I couldn't get past the video... At least someone saying, 'Oh yeah, we know it's a problem'—that's helpful…people just want to know. Telling people nothing is more infuriating." |
| Theme 6. Boosting Motivation for Behaviour Change: Trusted Experts and Personal Stories | **Expert-delivered information** | *"The first video told me what SYS and DIA meant. I wasn't really sure before…I was very interested to find out every day what my blood pressure was...I used to put salt on everything, but now I've stopped because of this."P3 (60-69y, Mixed)* |
|  | **Personal stories- Simplicity** | *“Probably it’s nothing new… the story was pretty simple. I mean, he (the character in the video) was told to join a club and go for a walk, get an interest. But it’s nicely expressed. It’s gentle and nice. It reminds the user that these things are very useful.” (P7, age group 70-79y White)* |
