# Supplementary figures and images for: Usability of the Coach-Supported Dementia Prevention App ENHANCE (Tailored Intervention for Brain Health and Cognitive Enrichment) in Older Adults: 1-Week Mixed Methods Study
Source: JMIR Aging. 2026 Jul 23;9:e92800. doi: 10.2196/92800 (PMC13395424; doi:10.2196/92800)

***Screenshot of Stump Shuffle***

***
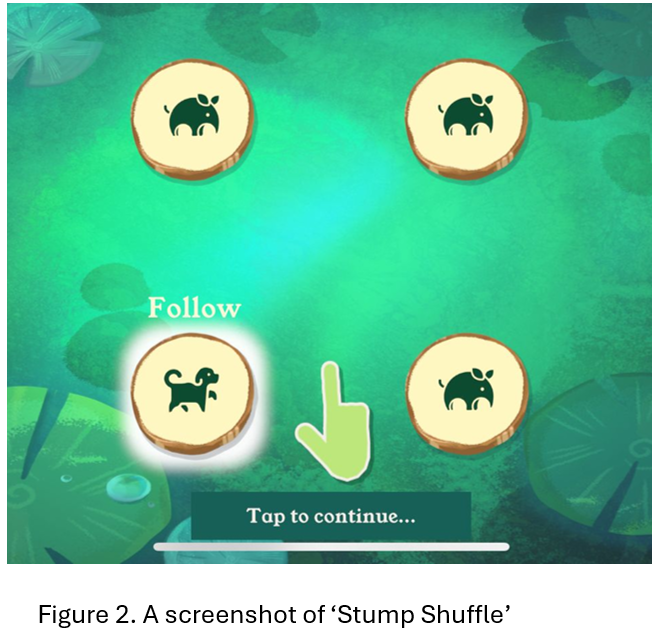
***

Supplement: Multimedia Appendix 6 [file aging-v9-e92800-s006.docx]
